# The effectiveness of massage interventions on procedural pain in neonates: A systematic review and meta-analysis

**DOI:** 10.1097/MD.0000000000030939

**Published:** 2022-10-14

**Authors:** Jiang Liu, Shirong Fang, Yuxia Wang, Lunan Gao, Tingting Xin, Yuxiu Liu

**Affiliations:** a School of Nursing, Weifang Medical University, Weifang, China; b Weifang People’s Hospital, Weifang Medical University, Weifang, China; c Weifang Maternal and Child Health Hospital, Weifang, China.

**Keywords:** massage, neonate, pain, non-, pharmacological interventions

## Abstract

The painful procedures experienced by neonates during hospitalization have short-term or long-term effects on neonates. While the limitations of previous interventions make it imperative to explore effective interventions that are readily available. This systematic review and meta-analysis was conducted to evaluate the safety and effectiveness of massage for pain management in neonates.

**Methods:**

This systematic review was registered in PROSPER. PubMed, Embase, Cochrane Library, and the Clinical Trials Registry were searched to December 2021. Two reviewers independently carried out study selection, data extraction, bias risk assessment. Continuous data were analyzed by mean differences (MD). Dichotomous data were reported using relative risk. If at least two studies reported identical results by the same pain assessment tool, a meta-analysis was conducted using random effect model and inverse variance.

**Results:**

Total 11 included studies involving 755 neonates investigated the effects of massage on neonatal pain response compared to standard care. The meta-analysis showed that massage could effectively improve pain response in neonates compared to standard care no matter whether neonatal infant pain scale (NIPS) or premature infant pain profile (PIPP) was used as an assessment tool. Besides, massage was also effective for crying duration, blood oxygen saturation both during and after the procedure, but non-effective for the variation of respiratory rate after the procedure, and heart rate both during and after the procedure.

**Conclusions:**

Massage may have a positive effect on pain relief of neonate, and rigorous trials are needed in the future to determine the most effective massage method.

## 1. Introduction

Neonates undergo many painful examinations in hospital. Each infant undergoes 7.5 to 17.3 painful examinations every day, such as heel-pricks, venipunctures, etc.^[1]^ Which undoubtedly brings great pain to newborns. It is reported that repeated pain examinations will cause short-term or long-term adverse consequences for neonates,^[2–5]^ such as reducing cerebral blood flow, hinder the development of neurological or motor functions and even cause hypersensitivity to pain.^[6–8]^ This highlights the significance of pain management for infants who experience painful procedures during hospitalization.

Although there are many pharmaceutical interventions that could be able to alleviate the pain of infants, the safety of long-term use remains to be studied.^[9]^ As a safe and reliable pain management method, non-pharmacological intervention is increasingly favored by parents of neonates.^[10,11]^ There are a growing body of researches on non-pharmacological interventions, such as, non-nutritive sucking, skin-to-skin contact, and breastfeeding before painful procedures. These interventions have been proved to be effective in alleviating pain to newborn babies.^[12–14]^ But these interventions require the presence of a mother or one of the parents, which is not always appropriate in a variety of clinical settings. So, we need to explore innovative interventions that can be used anytime in any setting and are effective for pain relief in newborns.

Effective interventions would ideally be inexpensive, noninvasive, and be rapidly applied to improve pediatric pain control.^[13]^ Massage, as an effective non-pharmacological intervention, has been gradually applied in various areas of clinical practice. It is a method applied by stimulating the acupuncture points and meridians in body using hands or special tools.^[15,16]^ Studies have shown that massage can relieve stress and improve blood circulation by reducing the levels of cortisol and increasing the levels of serotonin and dopamine.^[17,18]^ By stimulating the release of endorphins and serotonin, massage can relieve the pain of neonates, improve their sleep, and have a positive impact on the growth and development of neonates.^[19]^ Besides, massage can also activate the parasympathetic nervous system as a result of stimulation of the vagal nerves and provide calm and rest in the body.^[20]^

To our knowledge, only one systematic review on the non-pharmacological interventions^[11]^ that reported the effectiveness of massage in procedural pain relief of infants previously. However, in this review, the massage was found to be effective in alleviating the pain response of premature infants, but not for neonates. Other recent reviews reported that the massage was safe and effective for neonates’ pain relief.^[21]^ Moreover, a lot of studies have been conducted on the effects of massage for neonatal pain management in recent years, but the results were either controversial or partially effective.^[22–24]^

Therefore, a more comprehensive systematic review and meta-analysis is needed to assess the safety and effectiveness of this intervention in the pain management of preterm and full-term infants. The purpose of this study was to comprehensively evaluate the effectiveness of massage on pain relief and other secondary outcomes (the variation of heart rate, respiration, blood oxygen saturation, crying time, cortisol levels, and adverse events) in premature and full-term infants.

## 2. Methods

### 2.1. Protocol and registration

This systematic review protocol was registered in PROSPERO database (CRD42022302115). We conducted this systematic review according to the recommendations of preferred reporting items for systematic reviews and meta-analyses guidelines.^[25]^

### 2.2. Search strategy and selection criteria

We conducted electronic searches under the guidance of a library search specialist in the following databases to December 2021: PubMed, Embase, the Cochrane Library and the Clinical Trials Registry. Relevant articles were retrieved by combining the following medical subject headlines (MeSH) and keywords: (Infants OR Premature OR Preterm OR Neonatal OR Prematurity OR Newborn OR Neonate) AND (massage OR touch OR pain management).

Eligible studies included had to fulfill the following criteria: randomized controlled/clinical trials that conducted on the neonates with gestation between 24 and 42 weeks; studies comparing massage interventions (massage, or therapeutic touch) to comparator groups (offering standard care); with outcome including one of the ten most common painful procedures experienced by infants such as heel-prick, venipuncture et al^[26]^ Articles were excluded, if: reviews and case reports; studies not in English; studies without valid data or with improper data.

All standardized measurement scales and tools related to neonates’ painful evaluation would be considered, including: premature infant pain profile (PIPP)^[27,28]^ suitable for newborns between 28 and 40 weeks of gestation (WG); Neonatal Infant Pain Scale (NIPS) from 26 to 47 WG newborns^[29]^; neonatal facial coding system for 26 to 47 WG newborns.^[30]^ We combined different tools in our systematic review for pain assessment.

The main outcome was the neonates’ painful response, according to the suggestion of Pillai Riddell.^[11]^ Secondary outcomes included the variations of heart rate, blood oxygen saturation, respiration rate during and after painful examination and variations of the crying time, cortisol levels, occurrence of adverse events between before and after the painful procedure. Besides, the measurements taken within five minutes after the painful procedure were considered. As for measurements taken before discharge, we included the closest one to discharge.

### 2.3. Study selection and data extraction

We managed all the references in EndNote X9. After removing the duplication, two authors (LNG&TTX) independently reviewed the included studies according to the title and keywords, and then all eligible studies were retained for full-text assessment to determine whether suitable for inclusion in this systematic review. The reasons for the excluded references were recorded. To ensure the consistency of data, two reviewers extracted data independently and stored the data in Review Manager software. Before data analysis, two reviewers checked the data to avoid errors. The above process required two reviewers to reach a consensus, any dissenting opinions about the inclusion were resolved by consulting other reviewers (SRF&YXW).

### 2.4. Quality assessment

The Risk of Bias in the included studies was independently assessed by two reviewers (LNG&TTX) according to version 2 of the Cochrane risk-of-bias tool for randomized trials (RoB2) recommended by Cochrane Handbook for Systematic Reviews of Interventions, including bias arising from the randomization process, bias due to deviations, bias due to incomplete data, bias in measurement of the outcome, bias in selection of the reported result.^[31]^ The risk of bias for each study was classified as low risk of bias, high risk of bias, and some concerns.

### 2.5. Statistical analysis

Statistical analysis was conducted in the Review Manager software by using a random-effect model with a 95% confidence interval (CI). Since neonatal pain was assessed using different tools, we treated each tool separately to ensure data consistency. Continuous data were analyzed using mean differences (MD). Dichotomous data were reported using relative risk. If at least two studies reported identical results using the same pain assessment tool, a meta-analysis was conducted by using the random effect model and inverse variance. Subgroup analysis would be conducted according to the different massage method to provide further guidance for clinical practice. Using the chi-square test with a significance level of 0.1 to assess the heterogeneity of included studies. According to the suggestions of preferred reporting items for systematic reviews and meta-analyses-P,^[32]^ we classified I² as not important heterogeneity (0%–40%); moderate heterogeneity (30%–60%); substantial heterogeneity (50%–90%) and considerable heterogeneity (75%–100%).

## 3. Results

### 3.1. Study selection

**Figure 1.**
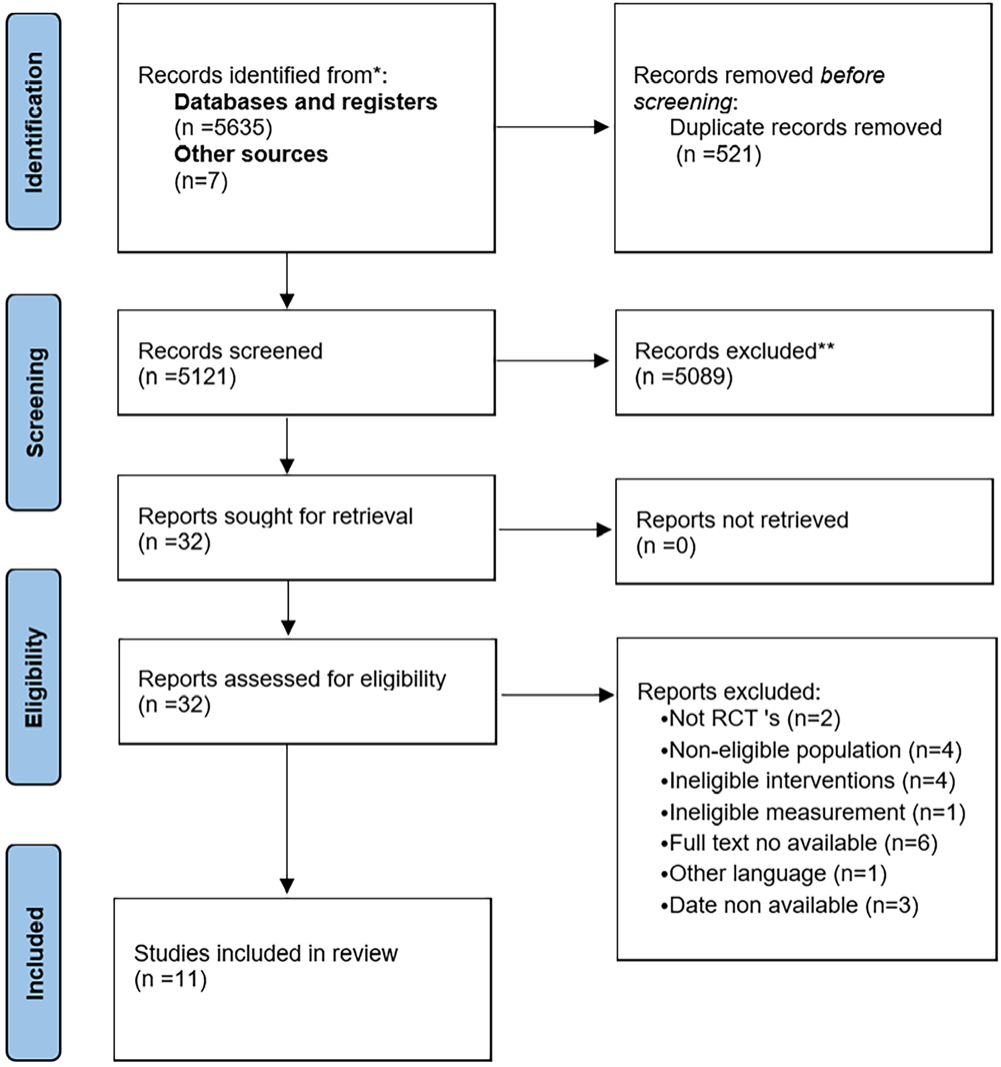
Preferred reporting items for systematic reviews and meta-analyses (PRISMA) Flow diagram of this meta-analysis.

The study selection process was showed by Flow Diagram (Fig. 1). A total of 5635 articles were identified through database searching, and 7 related studies were also included through snowballing, gray literature review and other methods. After the removal of 521 duplicated articles, a total of 5121 articles were retained. We eliminated 5089 results in the first screening by reading questions and keywords, and 32 studies were evaluated to be eligible for final selection (full-texts). In total, 21 articles were excluded for different reasons: 2 articles were not randomized controlled trials; 4 articles were not eligible population; 4 articles were not eligible interventions; 1 article was excluded for measurement; 6 articles were excluded because the full text could not be obtained even after contacting the authors; 1 article was not considered for language reasons; 3 articles’ dates could not be used. Finally, we conducted a meta-analysis of 11 studies, including 755 neonates investigating the effects of massage on neonatal pain response compared to standard care.^[17,33–42]^

### 3.2. Characteristics of included studies

The 11 studies were published between 2006 and 2021 written in English, and the characteristics were summarized in Table 1. They were carried out in 5 different countries, 2 in China,^[33,40]^ 4 in Iran,^[37,39,41,42]^ 1 in Korea,^[34]^ 2 in Canada,^[17,35]^ and 2 in Turkey.^[36,38]^ Five studies^[34,36–38,41]^ included neonates gestational age older than 37 weeks. Five studies^[17,35,39,40,42]^ included premature younger than 37 weeks of gestational age, and one study^[33]^ included newborns between 30 and 40 weeks of gestational age. All of the studies were randomized controlled trials, and the interventions studied were touch or massage. Seven of the studies were on massage^[17,33,36–39,41]^ and four were on therapeutic touching,^[34,35,40,42]^ but they were all included because the interventions were the same or similar. Moreover, three of the studies were cross-designed^[17,33,40]^ and the rest were parallel designed.

**Table 1:** Characteristics of included studies.

Study	Participants	Study design	Painful procedure	Intervention description	Outcome	Scale type	Timing of assessment	Main findings
Seçil Yavaş 2021	N = 128 full-term	RCT 2 parallel groups	Heel-prick	Massaged the baby’s foot for 3 minutes before the heel lancing procedure	Pain, Comfort Behavior Scale	NIPS	Before, during and three minutes after the procedure	NIPS:MG < CG
Turkey	Age = 37–42 weeks					NCBS		NCBS:MG < CG
Atefeh Roshanray 2020	N = 135 full-term	RCT,3 parallel groups	Hypothyroidism screening	Massaged the baby’s foot from the fingertips to the middle of the leg for 2 minutes before blood sampling	Pain,H R, RR, Sao2	NIPS	Before, immediately and 5 minutes after blood sampling	NIPS: MG < CG
Iran	Age = 38–42 weeks				Cry time			HR:MG < CG
								RR: MG > CG
								Sao2:MG < CG
								Cry time:MG < CG
Tuba Koç Özkan 2019	N = 139 full-term	left 3 parallel groups	Heel-left	Foot left for two minutes before heel lancing	Pain,HR, left	NIPS	During the heel lancing and 1 minute after heel lancing	NIPS: MG < CG
Turkey	Age >37 weeks				Cry time			HR:MG > CG
								Sao2: CG < MG
								cry time:MG < CG
Sunil Jain 2006	N = 23 Preterm	RCT 2 crossover groups	Heel-prick	Massaged the outer aspect of the leg chosen for the heel stick from toes to midthigh by using a firm but gentle pressure by fingers and thumbs before heel stick	Pain, HR, RR, Sao2, serum cortisol	NIPS	Before and after massage or no massage and 5 min after heel stick	NIPS:MG < CG
Canada	Age <37 weeks							HR:MG < CG
								RR:MG < CG
								Sao2:MG > CG
								Serum
								Cortisol:MG < CG
Hyesang Im 2008	N = 99 full-term	RCT 3 parallel groups	Heel-prick	Yakson (i.e. a traditional Korean touching method) were provided for 1min prior to starting the heel stick procedure and lasted until the completion of the procedure	Pain, HR, Sao2	NIPS	Before and 1 minute after heel lancing	NIPS:MG < CG
USA	Age = 37–42 weeks							HR:MG < CG
								The change of SaO2 levels in MG was lower than that of the CG
Gholami A 2021	N = 90 Preterm	RCT 3 parallel groups	Heel-prick	The massage was performed by her three times a day, five minutes each time	Pain	NIPS	Before discharge	NIPS:MG < CG
Iran	Age = 28–36 weeks							
Celeste Johnston 2013	N = 55 Preterm	RCT 2 parallel groups	Heel-prick	Therapeutic Touch for 5 minutes before heel lancing	Pain	PIPP	During the heel lancing	PIPP:MG > CG
Canada	Age = 26–32 weeks							
Yuen-Man Chik 2017	N = 80 Neonate	RCT 2 crossover groups	Venipuncture	Massage to both upper limbs for 2 minutes	Pain	PIPP	After venipuncture	PIPP:MG < CG
China	Age = 30–40 weeks							
Yongping Sun 2020	N = 66 Preterm	RCT 2 parallel groups	Screening for Retinopathy	The Gentle Human Touch protocol was implemented from the beginning of each procedure until 10 min after the procedure	Pain, HR, Sao2, rSCO2	PIPP	Before and during screening for Retinopathy	PIPP:MG < CG
China	Age <37 weeks							HR:MG > CG
								rSCO2:MG > CG
								Sao2:MG > CG
Mirzarahimi M 2013	N = 90 full-term	RCT 3 parallel groups	Heel-prick	Two minutes before the heel stick, one of the investigators slowly massaged the outer aspect of the leg chosen for the heel stick from toes to mid thigh by using a firm but gentle pressure by fingers and thumbs	Pain, HR, Sao2	PIPP	Before and after heel lancing	PIPP:MG < CG
Iran	Age >37 weeks							Sao2:MG > CG
								HR:MG < CG
Maryam Fatollahzade 2020	N = 34 Preterm	RCT 2 crossover groups	Endotracheal suctioning	The Gentle Human Touch protocol was implemented during the endotracheal suctioning	Pain	PIPP	After endotracheal suctioning	PIPP:MG < CG
Iran	Age <37 weeks							

NIPS = neonatal infant pain scale, PIPP = premature infant pain profile, RCT = randomized controlled trial, RR = risk ratios.

Included studies evaluated different painful procedures during hospitalization including venipuncture (n = 1),^[33]^ hypothyroidism screening (n = 1),^[37]^ heel-prick (n = 7),^[17,34–36,38,39,41]^ screening for retinopathy (n = 1),^[40]^ and endotracheal suctioning (n = 1).^[42]^

Different assessment tools were used in the included studies, but all were reliable and valid standard assessment scales. Six used the Neonatal Infant Pain Scale^[17,34,36–39]^ and five used Premature Infant Pain Profile.^[33,35,40–42]^ In addition to pain assessments, several second outcomes were also included in the study, including heart rate, respiration, oxygen saturation and crying time. Moreover, there were some differences in the assessment time between studies. Three studies^[24,43,44]^ were removed from the meta-analysis since the data was not able to be obtained, we reported their results through systematic synthesis.

### 3.3. Evaluation of evidence quality

**Figure 2.**
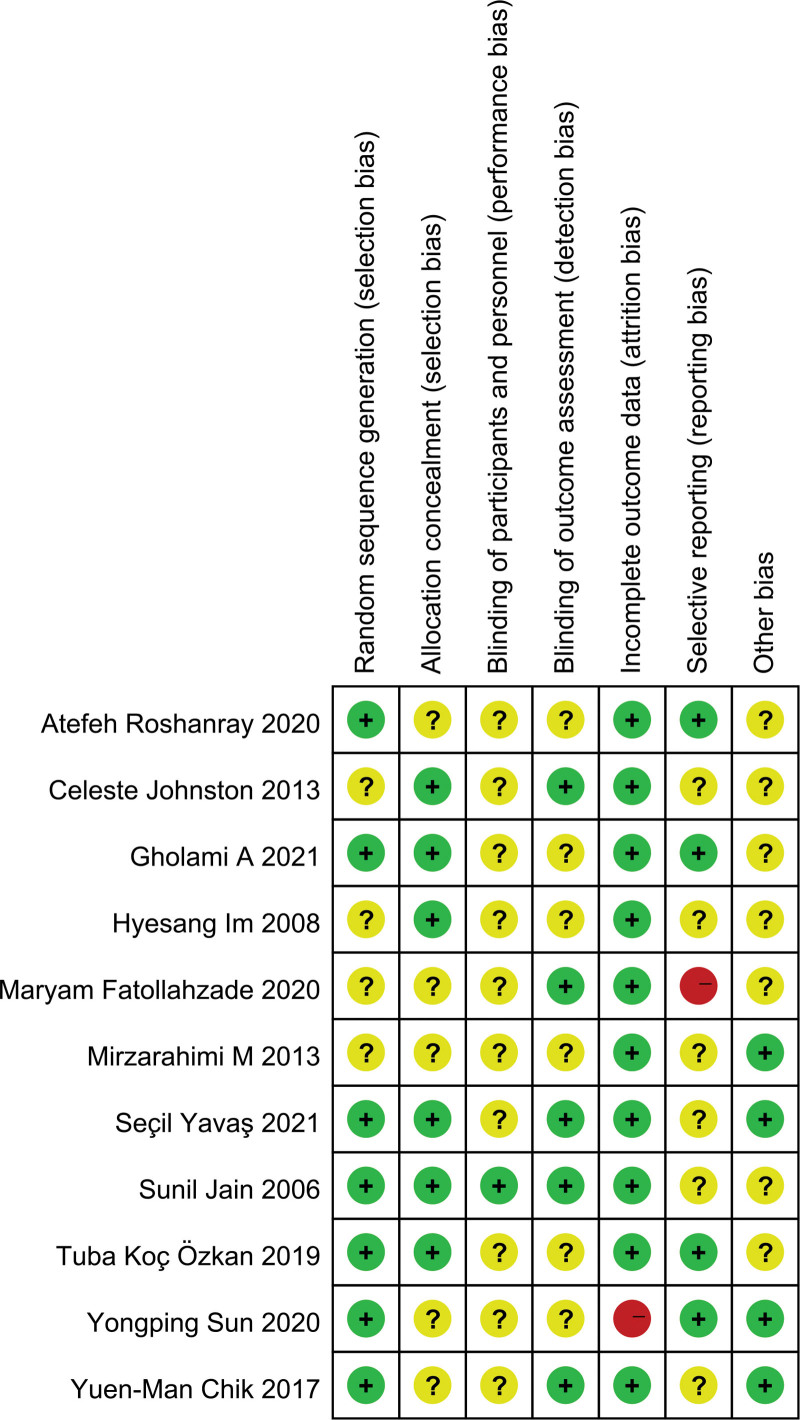
Risk of bias summary.

The risk on bias of each included study was presented in Fig. 2 and the detail was also summarized (see Supplemental Table S1, http://links.lww.com/MD/H461, Supplemental Content, which summarized the risk of bias in studies). Seven studies reported the methods of random-sequence generation detailedly,^[17,33,36–39,42]^ while others were unclear since insufficient information. Six of the studies described the detail of allocation concealment method appropriately,^[17,34–36,38,39]^ but the rest were assessed as unclear bias risk. All but one study were unclear risks since the nature of the intervention and the difficulty of blinding participants.^[33–42]^ For the blinding of outcome assessment, there were five studies describing it,^[17,33,35,38,40]^ while the rest were uncertain. The risk of incomplete outcome data was high in only one study,^[42]^ because the missing date of different groups was unbalanced and the reasons were different. For selective reporting, four studies^[36,37,39,42]^ were judged low but one^[40]^ was high risk since not all of the prespecified outcomes were reported. Of all the studies, we considered only four^[33,38,41,42]^ to be free from the bias of other sources, and others unclear for insufficient evidence provided.

### 3.4. Pain response score

**Figure 3.**
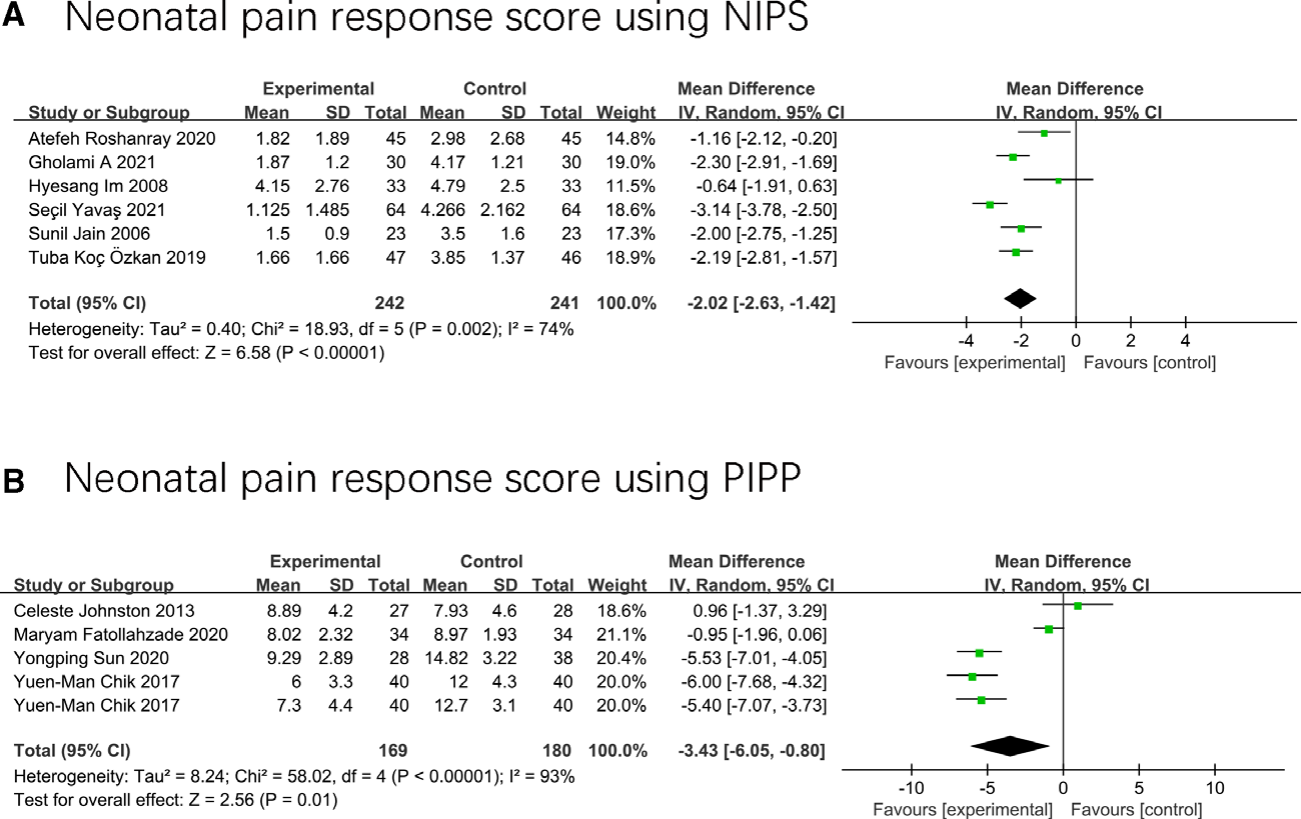
Forest plot displaying the results of pain response score.

Six studies^[17,34,36–39]^ including 460 neonates investigated neonatal pain response using NIPS and found that the massage was effective in improving neonatal pain response compared to standard care (MD −2.02; 95% CI −2.63 to −1.42; I^2^ = 74%; *P* < .01) (Fig. 3A).

**Figure 4.**
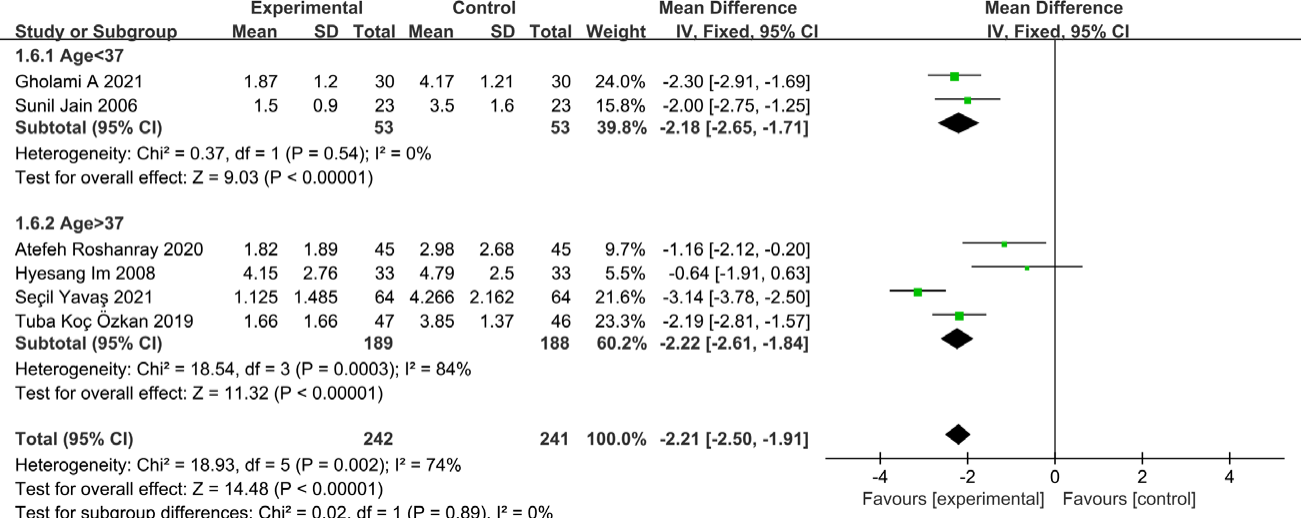
Forest plot displaying the results of subgroup analysis by gestational age of neonates.

According to subgroup analysis of age difference of neonates, massage could effectively improve their pain response regardless of whether the intervention group was full-term infants or premature infants. The differences between subgroups were not statistically significant (*P* = .89, I^2^ = 0%) (Fig. 4).

**Figure 5.**
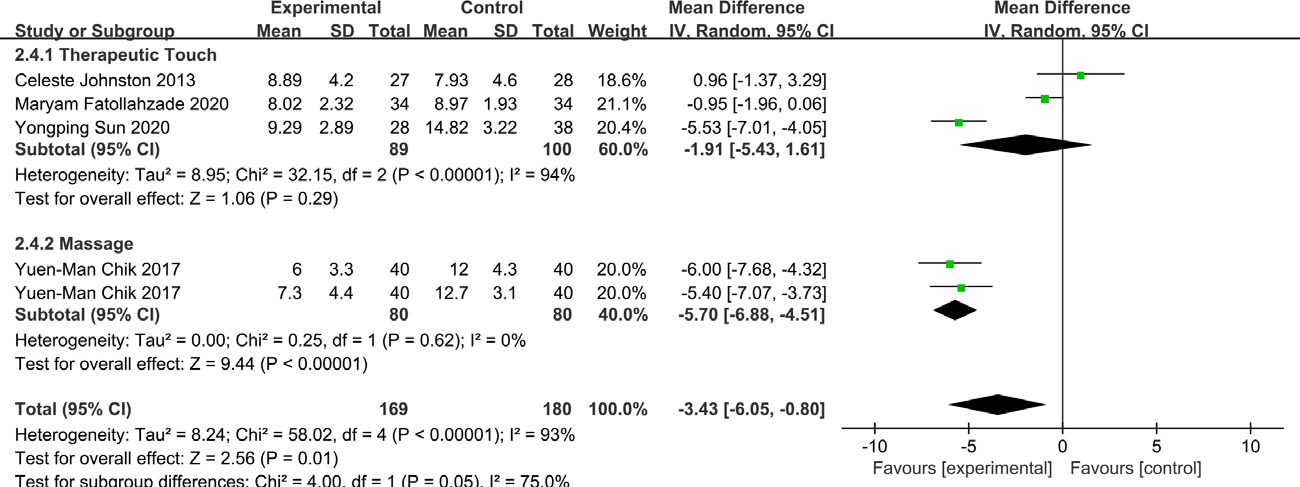
Forest plot displaying the results of subgroup analysis by different interventions.

Four studies^[33,35,40,42]^ including 235 neonates investigated the pain response of neonates using the PIPP scale and showed that the pain response scores of neonates who received massage prior to the pain procedure were significantly lower than those who received routine care (MD −3.43; 95% CI −6.05 to −0.80; I^2^ = 93%; *P* = .01) (Fig. 3B). According to different intervention measures, subgroup analysis found that the differences between subgroups were statistically significant (*P* = .05 I^2^ = 75%) (Fig. 5), and the massage was effective for neonates, while therapeutic touch was not effective for neonates.

### 3.5. Variation of heart rate

**Figure 6.**
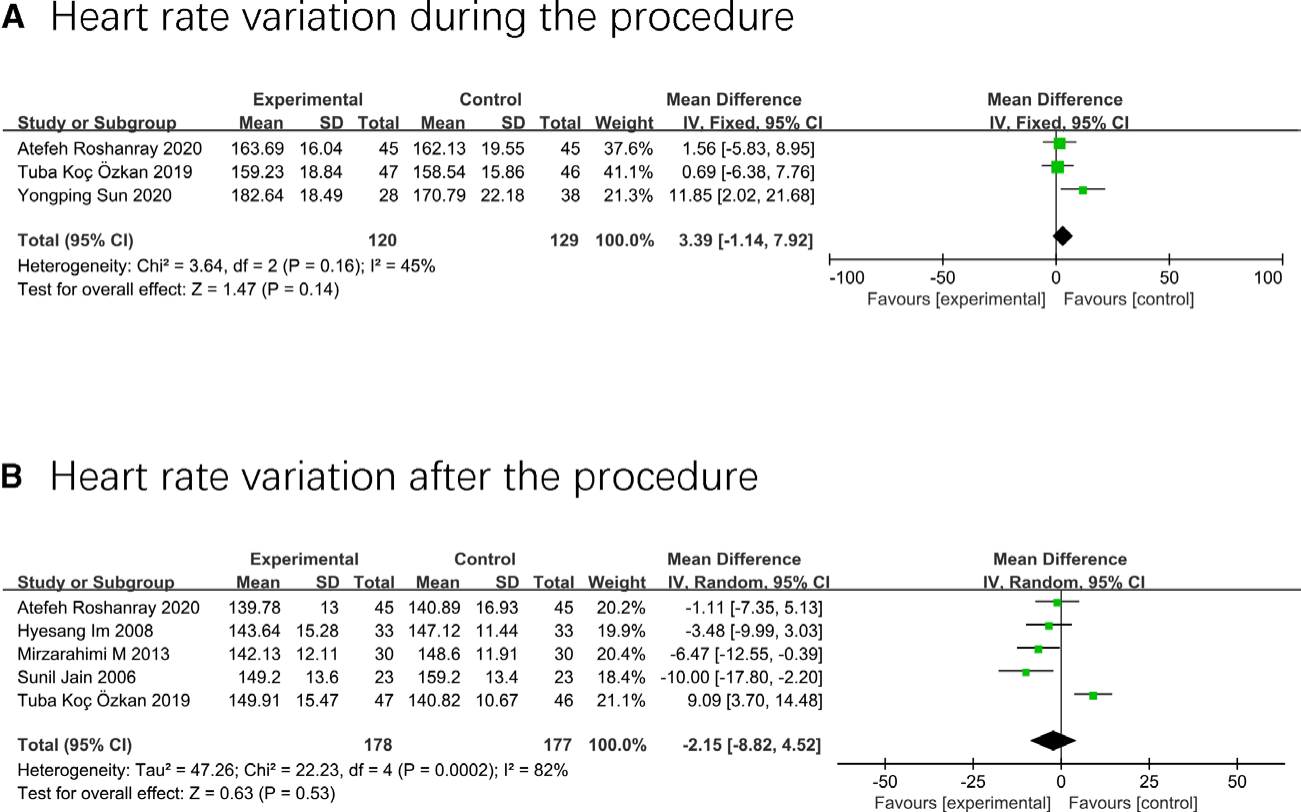
Forest plot displaying the results of heart rate.

Three studies (n = 249) investigated the variation of heart rate during the painful procedure,^[36,37,42]^ and there was no significant difference between the massage group and the standard care group (MD 3.39; 95% CI −1.14–7.92; I^2^ = 45%; *P* = .14) (Fig. 6A).

Five studies with 332 neonates^[17,34,36,37,41]^ analyzed the variations of heart rate after the painful examination, and the result showed that massage did not lower the heart rate of neonates after procedure compared to routine care (MD −2.15; 95% CI −8.82 to 4.52; I^2^ = 82%; *P* = .53) (Fig. 6B).

### 3.6. Variation of blood oxygen saturation

**Figure 7.**
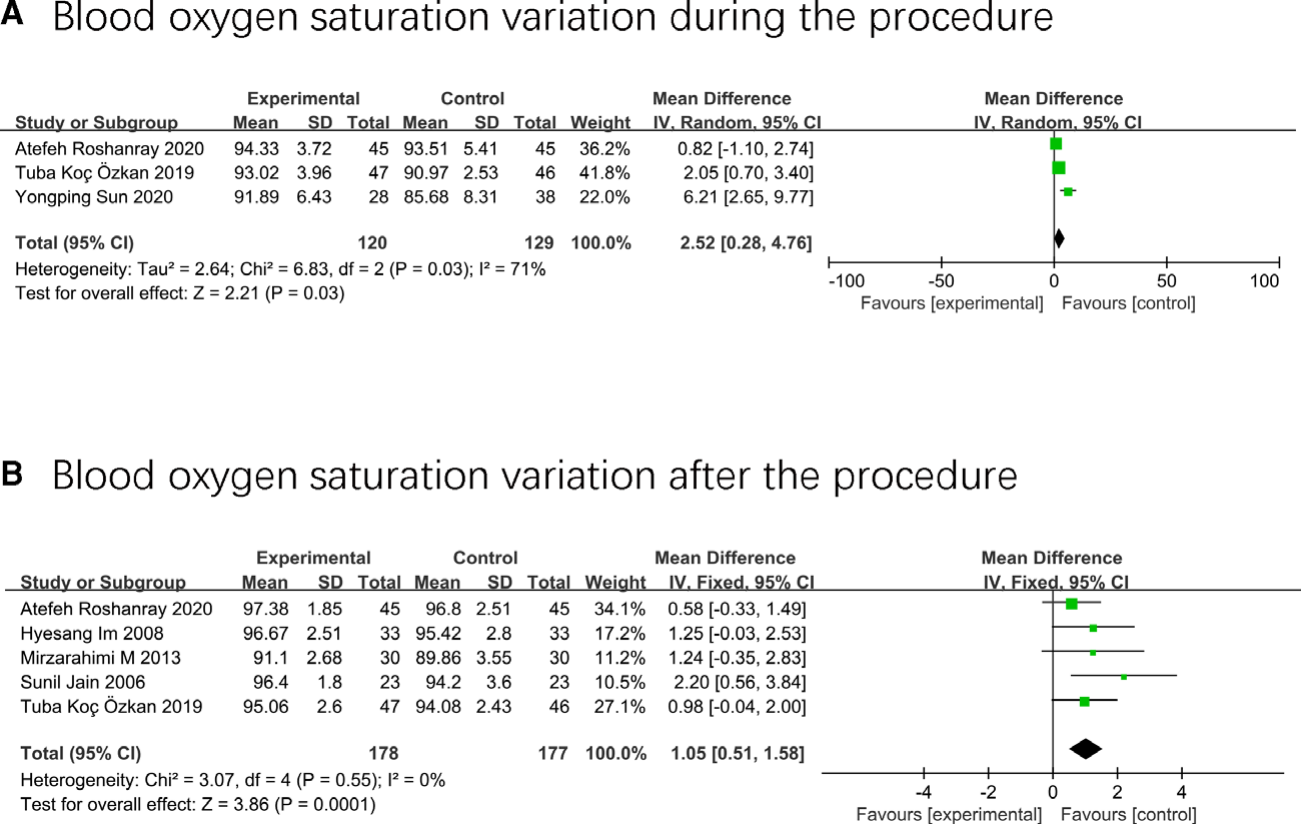
Forest plot displaying the results of blood oxygen saturation.

Three studies involving 249 neonates^[36,37,42]^ explored the effects of massage on the change of blood oxygen saturation during painful examination, and the merged result showed that massage was more effective than standard care in improving neonate blood oxygen saturation during the examination (MD 2.25; 95% CI 0.28–4.76; I^2^ = 71%; *P* = .03) (Fig. 7A).

Five studies including 332 participants^[17,34,36,37,41]^ revealed the massage group had a significant advantage over the control group in improving the blood oxygen saturation after the examination (MD 1.05; 95% CI 0.51–1.58; I^2^ = 0%; *P* < .01) (Fig. 7B).

### 3.7. Respiratory rate variation and duration of crying

**Figure 8.**
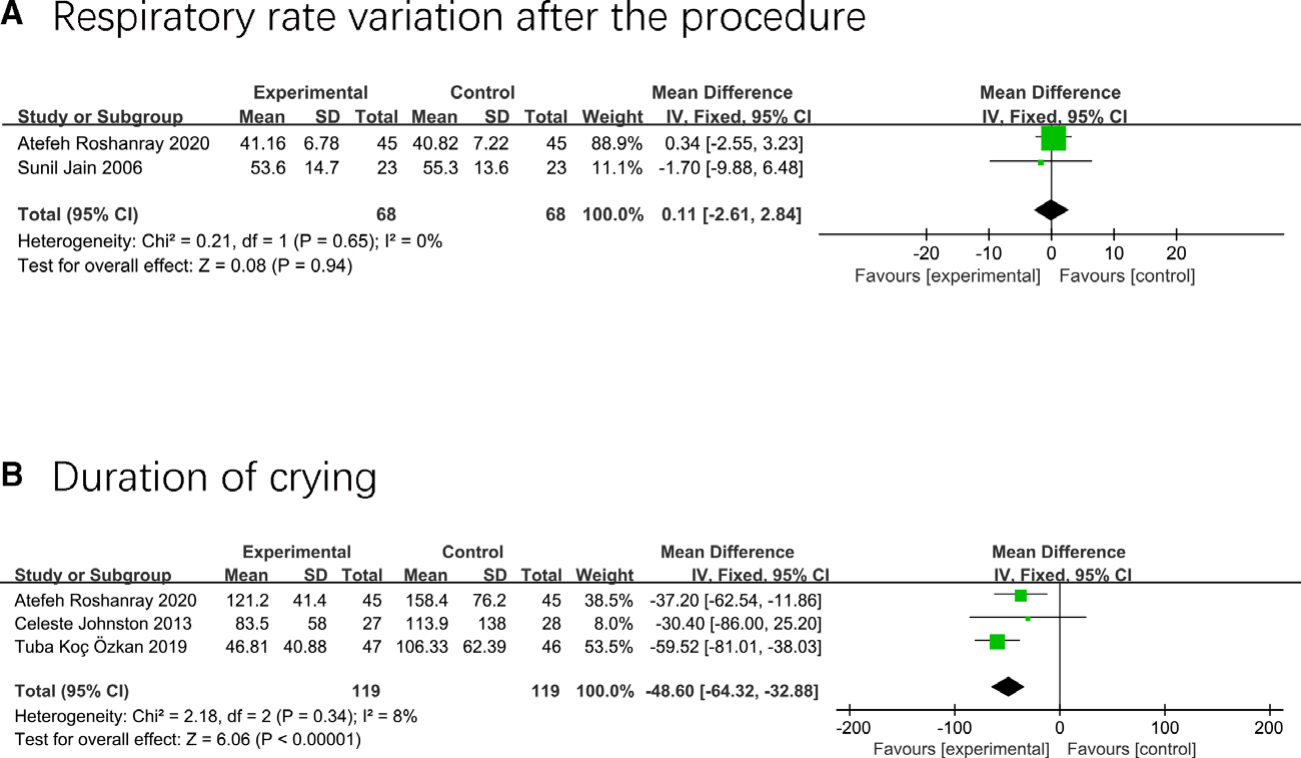
Forest plot displaying the results of respiratory rate and duration of crying.

Only two studies^[17,37]^ involving 113 neonates measured the effect of massage on respiration rate after neonatal procedure, and the results showed no effect on respiration rate (MD 0.11; 95% CI −2.61–2.84; I^2^ = 0%; *P* = .94) (Fig. 8A).

Three studies including 238 neonates^[35–37]^ revealed the effect of massage on crying time during painful procedures, and the merged result showed that neonates in the experimental group spent less time crying than those in the standard care group (MD −48.6; 95% CI −64.32 to −32.88; I ² = 8%; *P* < .01) (Fig. 8B).

## 4. Discussion

To our knowledge, this is the first systematic review and meta-analysis to examine the effectiveness of massage in relieving procedural pain in neonates during hospitalizations. We conducted all major and second outcomes of neonates’ pain response including neonates’ pain behaviors, heart rate, respiration, blood oxygen saturation, crying time, cortisol level and adverse events. Findings of 11 studies were synthesized in this review. For studies with the same measurement tools, they were combined for meta-analysis, if not, we conducted a separate evaluation in the form of narration. In the meta-analysis, we synthesized the results of 6 studies^[17,34,36–39]^ using NIPS, and found that massage had a significant effect on neonates’ pain relief compared to standard care. Subgroup analysis found that massage was effective for both full-term and premature neonate. There were 4 studies^[33,35,40,42]^ using PIPP to evaluate neonates for pain, and the meta-analysis results were the same. Subgroup analysis based on the different massage method showed that massage was more effective in relieving procedural pain than therapeutic touch.

In addition, we also found, through meta-analysis, massage had no effect on heart rate changes of neonates (during or after the procedure) compared with the control group. However, it had positive effects on blood oxygen saturation during and after the procedure. For crying time, neonates receiving massage had less crying time than neonates receiving standard care. However, in terms of respiration, we found massage had no significant effect on neonates.

For cortisol level, only one study reported that cortisol level in the experimental group had no significant differences compared to the control group.^[17]^ We could not conduct a meta-analysis on the occurrence of adverse events, because only a few authors explored the safety of massage, and relevant outcomes were not reported in all studies.

In the studies using NIPS, although the meta-analysis results showed that massage could effectively relieve the pain of neonates, the quality of evidence was not relatively high, which may be due to the clinical heterogeneity caused by differences in the different massage method and gestational age of neonates. We performed subgroup analysis based on whether the gestational age of neonates was greater than 37 weeks. The study found differences in gestational age did not influence the neonatal pain response (Fig. 4). While, in the study of neonates with gestational age less than 37 weeks, we consider the results stable and reliable, since the heterogeneity is small (*P* < .01, I² = 0%). In addition, we also found differences in the massage method across studies, which may cause clinical heterogeneity. But due to the lack of original studies, we were unable to do subgroup analyses based on differences in the massage method.

The results of subgroup analyses in studies using PIPP suggested the differences in the massage method (*P* = .05, I² = 75%) were likely to be the main source of heterogeneity. We also found the massage was more effective than therapeutic touch on neonatal pain relief. However, because of the insufficient number of original studies, we were unable to conduct subgroup analysis based on gestational age differences. There was still to be further strengthened in the original research. Noticeably, the different assessment tools were analyzed separately to reduce methodological heterogeneity caused by differences in assessment methods and improved the quality of evidence. In addition, analysis based on gender differences in future studies could help develop knowledge of the effectiveness of interventions.

As a non-pharmacological intervention, massage could be easily implemented at low cost and had few side effects.^[22]^ Although it may not be readily accessible for all NICUs, principles guiding non-pharmacological interventions, as massage, should still be encouraged to relieve painful reactions of neonate. The American Academy of Pediatrics considers it is necessary to combine various interventions of non-pharmacological to reinforce their effectiveness,^[9]^ and recent studies have found that multisensory stimulation had a positive effect on neonates in procedural pain relief.^[45]^ Massage can also be used in conjunction with other non-pharmacological interventions,^[46]^ such as olfactory stimulation and music therapy. Recent research found that multisensory stimulation had a significant effect on reducing procedural pain of neonate.^[47,48]^ Future studies may consider combining massage with other sensory interventions to evaluate the effectiveness of pain relief in neonates.

Our systematic review considered different pain assessment tools to evaluate massage for neonatal pain management during hospitalization. Although they are standard tools, there are some differences in measurement. In future studies, the combination of different tools is conducive to a more comprehensive assessment of the effectiveness of the intervention. One of the obvious problems for the pain assessment of neonate is the absence of a “gold standard”, and sometimes pain scales also can’t reflect painful response very well. Recently, one study showed that skin conductance (SC), as a physiological method, was an effective method in pain assessment of neonates. When pain occurs in neonates, the sympathetic nerve will respond to the stimulus. Skin conductance can evaluate pain by detecting changes in skin electrical activity and calculate SC values.^[49]^ Therefore, we suggest the measurement of SC should be combined with other standardized devices to be used in future neonatal pain measures.

### 4.1. Limitations

As fewer than 10 studies were included in our meta-analysis, we were unable to test the symmetry of funnel plot as originally planned. Our retrieval strategy only considered English literature. Although we contacted the authors in different ways, the missing data prevented us from conducting subgroup analysis. Moreover, there may be differences in the standard of care, which may affect our comparisons between studies.

## 5. Conclusions

In general, massage intervention plays a positive role in the relief of painful procedures in neonates, and we recommend it be used in clinical practice. Adverse events of massage intervention should be reported in future studies to guide clinical study and ensure the massage is carried out safely. In hospitals, especially in the NICU, neonates undergo a variety of painful procedures. So, it is imperative to identify the most effective interventions to manage procedural pain during hospitalization of neonate.

## Disclosure

The authors report no conflicts of interest in this work.

## Acknowledgments

The authors thank Weifang Health Commission Research Project for the funding on this review.

## Author contributions

**Conceptualization:** Jiang Liu, Yuxiu Liu.

**Data curation:** Jiang Liu, Shirong Fang, Lunan Gao, Tingting Xin.

**Formal analysis:** Jiang Liu, Lunan Gao.

**Funding acquisition:** Yuxia Wang, Yuxiu Liu.

**Investigation:** Shirong Fang, Yuxia Wang.

**Methodology:** Jiang Liu, Shirong Fang, Yuxia Wang, Lunan Gao.

**Resources:** Lunan Gao, Yuxiu Liu.

**Software:** Jiang Liu, Lunan Gao, Yuxiu Liu.

**Supervision:** Shirong Fang, Yuxia Wang, Yuxiu Liu.

**Validation:** Shirong Fang, Yuxia Wang, Tingting Xin.

**Visualization:** Shirong Fang, Yuxia Wang, Tingting Xin.

**Writing – original draft:** Jiang Liu.

**Writing – review & editing:** Yuxiu Liu.

## Supplementary Material


