# Supplementary material for: The effectiveness of massage interventions on procedural pain in neonates: A systematic review and meta-analysis
Source: Medicine (Baltimore). 2022 Oct 14;101(41):e30939. doi: 10.1097/MD.0000000000030939 (PMC9575769; doi:10.1097/MD.0000000000030939)
Supplement: Supplementary file 1 [file medi-101-e30939-s001.pdf]

Table S1. Bias Summary of Included Studies

Studies	Randomization (random sequence generation)	Allocation bias	Blinding of participants and personnel	Blinding of outcome assessment	Incomplete outcome data	Selective outcome reporting	Other potential sources of bias
**Seçil Yavaş 2021**	**Low risk of bias**	**Low risk of bias**	**Unclear risk of bias**	**Low risk of bias**	**Low risk of bias**	**Unclear risk of bias**	**Low risk of bias**
	"Randomization was thus provided by this method so that those who drew envelopes with"1"from the jar were included in the control group, and those who drew envelopes with"2"were in the intervention group."<br><br>The investigators describe a random component in the sequence generation process such as: Drawing of lots.	*"The item in the neonatal information form indicating that the"newborn is in the control or intervention group"was not marked. This item was marked after the procedure to ensure that the researcher did not know which group the newborn was in."*<br><br>Participants and investigators enrolling participants could not foresee assignment because one of the following, or an equivalent method, was used to conceal allocation: Sequentially numbered, opaque, sealed envelopes.	*The study did not address this outcome.*	*" The nurse then called the researcher back into the room. After the newborn was prepared for the procedure, the mother was asked to take her newborn in her arms, and the nurse duly conducted the heel lance. During the procedure, the newborn was evaluated by the investigator using the NIPS and NCBS."*<br><br>Blinding of outcome assessment ensured, and unlikely that the blinding could have been broken.	No missing outcome data reported.	The study protocol not published, and study not registered.	The study appears to be free of other sources of bias.
**Atefeh Roshanray**	**Low risk of bias**	**Unclear risk of bias**	**Unclear risk of bias**	**Unclear risk of bias**	**Low risk of bias**	**Low risk of bias**	**Unclear risk of bias**

**2020**							
	"Then using free online software, the randomization list was generated"<br><br>The investigators describe a random component in the sequence generation process such as: Referring to a random number table.	Insufficient information about the sequence generation process to permit judgement of 'Low risk' or 'High risk'.	The study did not address this outcome.	The study did not address this outcome.	No missing outcome data reported.	*The study protocol is registered (ID: IRCT2017011603197 2N4).*<br><br>All of the study's pre-specified outcomes have been reported.	The study appears to be free of other sources of bias.
**Tuba Koç Özkan 2019**	**Low risk of bias**	**Low risk of bias**	**Unclear risk of bias**	**Unclear risk of bias**	**Low risk of bias**	**Low risk of bias**	**Unclear risk of bias**
	"Cards written "acupressure", "massage" and "control" on them were placed in closed envelopes, and the mothers were asked to choose one of these envelopes."<br><br>The investigators describe a random component in the sequence generation process such as: Shuffling cards or envelopes.	*" Neonates were assigned in the acupressure, massage, and control groups with the closed envelope method."*<br><br>Participants and investigators enrolling participants could not foresee assignment because one of the following, or an equivalent method, was used to conceal allocation: Sequentially numbered, opaque, sealed envelopes.	Insufficient information to permit judgement of 'Low risk' or 'High risk'.	The study did not address this outcome.	Missing outcome data balanced in numbers across intervention groups, with similar reasons for missing data across groups.	*This study protocol is registered at ClinicalTrials.gov (NCT03906552).*<br><br>All of the study's pre-specified outcomes have been reported.	There may be a risk of bias, but insufficient information to assess whether an important risk of bias exist.

**Sunil Jain 2006**	**Low risk of bias**	**Low risk of bias**	**Low risk of bias**	**Low risk of bias**	**Low risk of bias**	**Unclear risk of bias**	**Unclear risk of bias**
	"Twenty five such envelopes had been prepared with the help of computer generated random numbers."<br><br>The investigators describe a random component in the sequence generation process such as: Using a computer random number generator.	*"Infants were randomised into two groups (massage first or massage second) by opening sequential sealed opaque envelopes."*<br><br>Participants and investigators enrolling participants could not foresee assignment because one of the following, or an equivalent method, was used to conceal allocation: Sequentially numbered, opaque, sealed envelopes.	*"Blinding of the caretakers to the massage or no-massage intervention was ensured by pulling the curtains around the bed until the intervention was completed."*<br><br>Blinding of outcome assessment ensured, and unlikely that the blinding could have been broken.	*"All the parameters were recorded by the bedside nurse who was blinded to the intervention."*<br><br>Blinding of outcome assessment ensured, and unlikely that the blinding could have been broken.	*"Data of three infants could not be included because two were transferred to other hospitals before completion of the study and one was later discovered to have received morphine within 24 h prior to initiation of the study."*<br><br>Missing outcome data balanced in numbers across intervention groups, with similar reasons for missing data across groups.	The study protocol not published, and study not registered.	The study appears to be free of other sources of bias.
**Hyesang Im 2008**	**Unclear risk of bias**	**Low risk of bias**	**Unclear risk of bias**	**Unclear risk of bias**	**Low risk of bias**	**Unclear risk of bias**	**Unclear risk of bias**
	The study did not address this outcome.	*"To randomly allocate a hospital to a group, three sealed envelops were prepared and one personnel of each hospital picked one envelop upon the hospital's decision to participate in the study."*<br><br>Participants and	Unclear if the nurse was aware of the research goal and unclear information as to whether mothers were blinded to intervention.	Insufficient information to permit judgment of 'Low risk' or 'High risk'.	No missing outcome data reported.	The study protocol not published, and study not registered.	There may be a risk of bias, but insufficient information to assess whether an important risk of bias exist.

		investigators enrolling participants could not foresee assignment because one of the following, or an equivalent method, was used to conceal allocation: Sequentially numbered, opaque, sealed envelopes.					
**Gholami A 2021**	**Low risk of bias**	**Low risk of bias**	**Unclear risk of bias**	**Unclear risk of bias**	**Low risk of bias**	**Low risk of bias**	**Unclear risk of bias**
	"Random allocation sequence was performed by the software."<br><br>The investigators describe a random component in the sequence generation process such as: Random number generation software.	*"Each allocation was sealed in a white envelope and numbered."*<br><br>Participants and investigators enrolling participants could not foresee assignment because one of the following, or an equivalent method, was used to conceal allocation: Sequentially numbered, opaque, sealed envelopes.	Insufficient information to permit judgment of 'Low risk' or 'High risk'.	Insufficient information to permit judgment of 'Low risk' or 'High risk'.	No missing outcome data reported.	*"The study protocol is registered (ID: IRCT2018010803826 5N2)."*<br><br>All of the study's pre-specified outcomes have been reported.	There may be a risk of bias, but insufficient information to assess whether an important risk of bias exist.

**Celeste Johnston 2013**	**Unclear risk of bias**	**Low risk of bias**	**Unclear risk of bias**	**Low risk of bias**	**Low risk of bias**	**Unclear risk of bias**	**Unclear risk of bias**
	Insufficient information to permit judgment of 'Low risk' or 'High risk'.	*"only the nurse providing the intervention had access to the Web site section containing the group assignment. The code was revealed to the primary author only after all analyses had been conducted."*<br><br>Allocation concealment ensured, and unlikely that the method could have been broken.	Insufficient information to permit judgment of 'Low risk' or 'High risk'.	*"The therapist drew the curtains around the incubator of the infant and either began Therapeutic Touch or sham Therapeutic Touch. After approximately 5 minutes, she left the curtained area, then the staff performing the heel lance and the research nurse entered the area and collected the required blood, with the research nurse marking the recordings with signals."*<br><br>Blinding of outcome assessment ensured, and unlikely that the blinding could have been broken.	*"Blood work was not required during study time (n = 9), infant was withdrawn by medical staff (n=2), data were missing from 1 infant and 1 infant was withdrawn because of becoming unstable during the study session."*<br><br>Reasons for missing outcome data unlikely to be related to true outcome (for survival data, censoring unlikely to be introducing bias).	The study protocol not published, and study not registered.	The study appears to be free of other sources of bias.
**Yuen-Man Chik 2017**	**Low risk of bias**	**Unclear risk of bias**	**Unclear risk of bias**	**Low risk of bias**	**Low risk of bias**	**Unclear risk of bias**	**Low risk of bias**
	"Eligible infants were randomly assigned to two groups in different order to receive the interventions using a table of random	The method of concealment is not described or not described in sufficient detail to allow a definite	Insufficient information to permit judgment of 'Low risk' or 'High risk'.	*"Ten physicians who did the venipuncture with the same technique and four trained nurses as pain score raters were blinded to the*	*"80 infants participated in the study, with 15 being eliminated due to not needing a second blood*	The study protocol not published, and study not registered.	The study appears to be free of other sources of bias.

	numbers: the massage first (Massage 1st) group or the massage second (Massage 2nd) group."<br><br>The investigators describe a random component in the sequence generation process such as: Referring to a random number table.	judgement.		*intervention."*<br><br>Blinding of outcome assessment ensured, and unlikely that the blinding could have been broken.	*sampling."*<br><br>Missing outcome data balanced in numbers across intervention groups, with similar reasons for missing data across groups.		
**Yongping Sun 2020**	**Low risk of bias**	**Unclear risk of bias**	**Unclear risk of bias**	**Unclear risk of bias**	**High risk of bias**	**Low risk of bias**	**Low risk of bias**
	"Using the random number table, we assigned 41 pre-term infants to each group."<br><br>The investigators describe a random component in the sequence generation process such as: Referring to a random number table.	The method of concealment is not described or not described in sufficient detail to allow a definite judgement.	The study did not address this outcome.	Insufficient information to permit judgment of 'Low risk' or 'High risk'.	Missing outcome data imbalanced in numbers across intervention groups, with different reasons for missing data across groups.	*"The study protocol is registered (ID: ISRCTN10976481)."*<br><br>All of the study's pre-specified outcomes have been reported.	*The study appears to be free of other sources of bias.*
**Mirzarahimi M 2013**	**Unclear risk of bias**	**Unclear risk of bias**	**Unclear risk of bias**	**Unclear risk of bias**	**Low risk of bias**	**Unclear risk of bias**	**Low risk of bias**
	Insufficient information about the sequence generation process to permit judgement	The method of concealment is not described or not described in sufficient detail to	The study did not address this outcome.	The study did not address this outcome.	No missing outcome data reported.	The study protocol not published, and study not registered.	There may be a risk of bias, but insufficient information to assess whether an important risk of bias exist.

	of ‘Low risk’ or ‘High risk’.	allow a definite judgement.					
**Maryam Fatollahzade 2020**	**Unclear risk of bias**	**Unclear risk of bias**	**Unclear risk of bias**	**Low risk of bias**	**Low risk of bias**	**High risk of bias**	**Unclear risk of bias**
	Insufficient information about the sequence generation process to permit judgement of ‘Low risk’ or ‘High risk’.	*The study did not address this outcome.*	*"All suctioning (both by the routine method and by gentle human touch) was performed by the same nurse."*<br><br>Unclear if the nurse was aware of the research goal and unclear information as to whether mothers were blinded to intervention.	*"For the purpose of blinding, touch samples were scored by a researcher familiar with the PIPP, and the samples were suction routinely scored by another researcher familiar with the instrument."*<br><br>Blinding of outcome assessment ensured, and unlikely that the blinding could have been broken.	*" 2 were excluded from the study due to Parental dissatisfaction with intervention, and 3 due to changes in treatment and intranasal tube use, and one neonate due to transfer to another hospital."*<br><br>Reasons for missing outcome data unlikely to be related to true outcome (for survival data, censoring unlikely to be introducing bias).	*The study protocol is registered (ID: IRCT20180508039587N1)*<br><br>Although cited as primary outcomes in protocol, no data were reported on variation of heart rate and oxygen saturation.	The study appears to be free of other sources of bias.

Note: Italics shows direct quotes from articles.
